# Event-related brain potential correlates of prospective memory in symptomatically remitted male patients with schizophrenia

**DOI:** 10.3389/fnbeh.2015.00262

**Published:** 2015-09-30

**Authors:** Guoliang Chen, Lei Zhang, Weiyan Ding, Renlai Zhou, Peng Xu, Shan Lu, Li Sun, Zhongdong Jiang, Huiju Li, Yansong Li, Hong Cui

**Affiliations:** ^1^215th Clinical Division, 406th Hospital of PLADalian, China; ^2^Department of Psychology, School of Social and Behavioral Sciences, Nanjing UniversityNanjing, China; ^3^The Research Center for Social and Behavioral Sciences of Jiangsu ProvienceNanjing, China; ^4^The Department of Neurology and Psychiatry, The First Affiliated Hospital of Dalian Medical UniversityDalian, China; ^5^Department of Medical Psychology, General Hospital of PLABeijing, China

**Keywords:** prospective memory, ERPs, symptomatically remitted patients with schizophrenia, N300, prospective positivity

## Abstract

Prospective memory (PM) refers to the ability to remember to perform intended actions in the future. Although PM deficits are a prominent impairment in schizophrenia, little is still known about the nature of PM in symptomatically remitted patients with schizophrenia. To address this issue, event-related brain potentials (ERPs) were recorded from 20 symptomatically remitted patients with schizophrenia and 20 healthy controls during an event-based PM paradigm. Behavioral results showed that symptomatically remitted patients with schizophrenia performed poorly on the PM task compared with healthy controls. On the neural level, the N300, a component of the ERPs related to PM cue detection, was reliable across these two groups, suggesting a degree of functional recovery of processes supporting cue detection in patients with symptomatically remitted schizophrenia. By contrast, the amplitude of the prospective positivity, a component of the ERPs related to PM intention retrieval, was significantly attenuated in symptomatically remitted schizophrenia patients relative to healthy controls. Furthermore, a significant positive correlation between the amplitude of the prospective positivity and accuracy on the PM task was found in those patients, indicating that patients’ poor performance on this task may result from the failure to recover PM cue-induced intention from memory. These results provide evidence for the existence of altered PM processing in patients with symptomatically remitted schizophrenia, which is characterized by a selective deficit in retrospective component (intention retrieval) of PM. Therefore, these findings shed new light on the neurophysiological processes underlying PM in schizophrenia patients during clinical remission.

## Introduction

Patients with schizophrenia are characterized by cognitive deficits (Heinrichs and Zakzanis, 1998; Elvevåg and Goldberg, 2000). Specifically, previous research has shown that schizophrenia patients show deficits in a variety of cognitive domains, including attention, executive function and memory (Chan et al., 2004; Mesholam-Gately et al., 2009). In the context of such generalized cognitive deficits, some researchers argue that memory deficit is one of the core impairments in those patients (Chan et al., 2004; Reichenberg and Harvey, 2007).

Prospective memory (PM) is thought to be an important aspect of episodic memory, which refers to remembering to perform intended actions in the future such as remembering to put the trash in a garbage bin at 9 am or to post a letter after seeing a mailbox (Meacham and Singer, 1977; Kliegel et al., 2008). Given that PM is so highly prevalent in our daily life, understanding its underlying mechanisms has gradually become a focus of experimental inquiry in cognitive psychology over the past decades (Graf and Uttl, 2001; Brandimonte et al., 2014). According to a popular conceptual framework, the realization of delayed intentions entails both prospective (cue detection) and retrospective (intention retrieval) components (McDaniel and Einstein, 1992; Smith and Bayen, 2004; Brandimonte et al., 2014). The prospective component refers to processes that support the detection of prospective cues (e.g., the post office) signaling that an intended action (e.g., buying a stamp) should be performed and the retrospective component involves processes that support subsequent recovery of that intention (e.g., buying a stamp) from memory (Einstein and McDaniel, 1990; Brandimonte et al., 2014). Moreover, work using event-related brain potentials (ERPs) has revealed that two ERP components (N300 and prospective positivity) are differentially related to the prospective and retrospective components of PM in humans (West et al., 2001; West and Ross-Munroe, 2002; West and Krompinger, 2005; West, 2011). The theoretical and empirical advances on PM also have clinical implications for exploring the nature of PM in patients with mental disorders (Rendell and Henry, 2009; Zogg et al., 2012).

Particularly, PM deficits in schizophrenia patients have been gaining increasing attention over the past decade. Earlier studies have revealed that schizophrenia patients performed more poorly than healthy controls across different types of PM tasks (event-based, time-based and activity-based PM; Elvevåg et al., 2003; Shum et al., 2004; Kumar et al., 2005, 2008; Altgassen et al., 2008; Ungvari et al., 2008). This is corroborated by recent meta-analytic studies describing that such deficits in schizophrenia patients can be observed with moderate to large effect sizes (Wang et al., 2009). Meanwhile, other recent research contributes to clarifying the nature and extent of PM deficits by controlling for other cognitive functions such as working memory (Zhuo et al., 2013), retrospective memory (Shum et al., 2004; Xiang et al., 2010), IQ (Lui et al., 2011), executive function (Henry et al., 2007; Lui et al., 2011) as well as clinical variables such as duration of illness (Zhou et al., 2012) and medication (Zhuo et al., 2013). These findings suggest that PM deficits reflect a primary impairment of schizophrenia rather than a secondary to other cognitive impairments (Henry et al., 2007; Wang et al., 2008; Ordemann et al., 2014). In addition, some researchers have begun to elucidate whether PM deficits are driven by impairments in specific components of PM in schizophrenia patients. The existing evidence suggests that schizophrenia-associated PM deficits are due to failures of both prospective (cue detection) and retrospective (intention retrieval) components (Woods et al., 2007; Wang et al., 2008).

Despite such evidence, little is still known about PM in symptomatically remitted patients with schizophrenia, although prior studies have shown the persistence of neuropsychological deficits in some cognitive domains including theory of mind (Mo et al., 2008) and emotional reaction (Yalcin-Siedentopf et al., 2014) in those patients. To address this question, we combined an event-based PM paradigm with ERPs in 20 symptomatically remitted patients with schizophrenia and 20 healthy controls. In this event-based PM task, participants were required to remember to respond to a word in blue (PM task), which was embedded in a simple categorization task (ongoing task). It is recognized that ERP-based biomarkers provide sensitive and reliable measures of the neural events underlying cognition. In particular, the promise of ERP-based biomarkers of cognitive dysfunction in schizophrenia has recently been demonstrated (Luck et al., 2011), thereby providing a unique opportunity to distinguish and examine the influence of processes associated with prospective (PM cue detection, N300) and retrospective (PM intention retrieval, prospective positivity) components of PM on task performance in symptomatically remitted patients with schizophrenia.

## Materials and Methods

### Participants

We recruited 20 symptomatically remitted patients with schizophrenia. All were right-handed males, because recruiting males could avoid the potential effect of the menstrual cycle known to have an impact on memory in females (Phillips and Sherwin, 1992; Wizeman, 2012). All patients had an earlier acute episode of schizophrenia according to DSM-IV-TR (Diagnostic and Statistical Manual of Mental Disorders (fourth edition, text revision)) criteria for schizophrenia diagnosis (Association, 2000). Diagnosis was determined by the Structured Clinical Interview for DSM-IV-TR AXIS I Disorders (SCID; First et al., 2001). Patients with a history of any past or present major medical or neurological illnesses, brain injury, drug dependence and mental retardation were excluded (according to medical records, information collected from family members and interview with the patients). Furthermore, those with schizoaffective, anxiety and depression disorders were excluded. We used the Chinese version of Positive and Negative Symptom Scale (PANSS; Kay et al., 1987) to measure severity of symptoms (Table 1). Symptomatic remission were defined according to a criteria (both severity of core symptoms and their time criteria) proposed by the Remission in Schizophrenia Working Group (RSWG; Andreasen et al., 2005). We also assessed the patients’ intellectual functioning using the Chinese version of the Wechsler Adult Intelligence Scale-Revised (WAIS-R; Gong, 1992), which has been employed to rate this function of Chinese schizophrenia patients in previous studies (Wang et al., 2008; Zhou et al., 2012).

**Table 1:** **Demographic and clinical characteristics of the study sample**.

	Remitted schizophrenia (*n* = 20) (M ± SEM)	Healthy controls (*n* = 20) (M ± SEM)	Group comparison
Age (years)	23.65 ± 0.58	22.55 ± 0.80	*t*_(38)_ = 1.11, *p* > 0.05
Education (years)	10.35 ± 0.57	10.65 ± 0.43	*t*_(38)_ = 0.42, *p* > 0.05
IQ	89.95 ± 2.78	98.05 ± 2.65	*t*_(38)_ = 2.11, *p* < 0.05
Duration of illness (months)	27.15 ± 4.01
Medication^a^	250.00 ± 28.89
PANSS^b^
Positive symptoms	8.80 ± 0.41
Negative symptoms	13.65 ± 0.77
General psychopathology	26.00 ± 1.07
Total score	48.45 ± 1.42

*M, mean; SEM, standard error of mean; ^a^Chlorpromazine equivalence mg/d; ^b^PANSS, Positive and Negative Symptom Scale*.

We also recruited 20 healthy controls reporting no prior history of psychiatric illness and drug abuse. Healthy controls also underwent psychiatric evaluation. Both groups were matched in age, gender and education (Table 1). A diagnosis of schizophrenia or any other mental disorders in first-degree relatives was also an exclusion criterion.

The study was carried out in according with the guidelines approved by the ethics committee of PLA general hospital. All participants in this study gave written informed consent to participate in the experiment.

### Stimuli and Event-Based PM Paradigm

The event-based PM paradigm followed the task used in a previous study (Zhuo et al., 2013). The ongoing task consisted of a simple categorization task. On ongoing activity trials, a Chinese word in green was presented in the center of a computer screen, while four different Chinese words in black referring to four different categories (animal, plant, commodity and people) were shown below the center of the screen. The participants’ task was to decide which category the word in green belongs to. In the PM task, the participants were asked to suspend the ongoing task and switch to the PM task by pressing a corresponding button on the Joystick when a word in blue (PM cue) was presented.

There were a total of 1000 trials which were equally divided into 10 blocks. Each block included 94 ongoing activity trials and 6 PM cue trials. Within each block, a stimulus (a word in green or blue), together with the words in black referring to four categories which maintained below the center of the screen throughout the entire block, was shown in the center of the screen and the participants had a maximum of 4000 ms to make a response indicating which category this word belongs to. After the response, the stimulus was replaced by a fixation cross lasting randomly for 500–800 ms. It was followed by the next stimulus within the block. The PM and ongoing trials were randomly interspersed within each block. Before the experimental task began, the participants performed a practice block containing 20 ongoing activity trials and 3 PM cue trials to ensure that they understood the instructions.

Words were drawn from text books and reading materials used in Chinese primary schools (People’s Education Press) and were matched for written frequency, familiarity, and concreteness. Words from four categories (animal, plant, commodity and people) were included. There were a total of 500 words and each category included 125 words. In each block, 25 words were randomly selected from each category.

### ERP Data Recording and Analysis

EEG was recorded (SynAmps amplifier, NeuroScan) with a quick cap carrying 32 Ag/AgCl electrodes (Fp1, Fp2, F3, Fz, F4, Fc3, Fcz, Fc4, C3, Cz, C4, CP3, CPz, CP4, P3, Pz, P4, F7, F8, Ft7, Ft8, T3, T4, Tp7, Tp8, T5, T6, O1, Oz, O2) placed at standard locations covering the whole scalp (the extended international 10–20 system). The reference electrode was attached to the right mastoid (A2), and the ground electrode was placed on the forehead. The vertical electrooculogram (VEOG) was recorded with electrodes placed above and below the left eye. The horizontal electrooculogram (HEOG) was recorded with electrodes placed beside the two eyes. The impedance was kept below 5 kΩ. The electrophysiological data were continuously recorded with a bandwidth 0.05–100 Hz and sampled at a rate of 1000 Hz.

Offline data analysis was conducted using EEGLAB (Delorme and Makeig, 2004) and ERPLAB (Lopez-Calderon and Luck, 2014). Data were first re-referenced to linked mastoid (A1 and A2). An independent component analysis (ICA)-based artifact correction was achieved by using the ICA function of EEGLAB. Independent components with topographies representing saccades, blinks, and heart rate artifact were thus removed according to published guidelines (Jung et al., 2000). The resultant EEG data were then epoched from 200 ms pre-stimulus to 1000 ms post-stimulus and digitally low pass filtered by 30 Hz (24 dB/octave). The 200 ms pre-stimulus period was used for baseline correction. In order to remove movement artifacts, epochs were rejected when fluctuations in potential values exceeded ±75μV at any channels except the EOG channel. The ERPs evoked by PM cue trials and ongoing activity trials were calculated by averaging individual artifact-free trials in each participant. Finally, the grand-averaged ERPs were computed and averaged for correctly performed PM cue trials and correctly performed ongoing activity trials in each group.

### Statistical Analysis

For statistical analysis on the behavioral data, both response times and accuracy were analyzed using a two-way mixed Analysis of Covariance (ANCOVA) with group (symptomatically remitted patients with schizophrenia vs. healthy controls) as a between-subject factor, task type (PM vs. ongoing task) as a within-subject factor. Because there was a significant between-group difference in IQ, we also included IQ in our mixed model as a covariate.

With regard to statistical analysis on electrophysiological data, our data were analyzed according to the topographical distribution of grand averaged ERP activity as well as the methods of previous ERP studies (West et al., 2001; West and Ross-Munroe, 2002). Our ERP statistical analysis involved two PM-related ERP components: N300 and prospective positivity. The N300 referred to the maximum negative voltage over the occipital region between 190 and 400 ms and the prospective positivity represented the maximum positive voltage over the parietal region between 400 and 1000 ms. Based on the methods of previous ERP studies (West et al., 2001; West and Ross-Munroe, 2002), the amplitudes of both ERP components were quantified as the mean voltages across 50 ms window that centered on the peaks so that the measurements would have similar levels of stability. Then, amplitudes of these two ERP components were analyzed using a three-way mixed ANCOVA with task type (PM vs. ongoing task) and electrode sites as within-subject factors, group (symptomatically remitted patients with schizophrenia vs. healthy controls) as a between-subject factor and with IQ as a covariate. The selection of electrodes characterizing N300 and prospective positivity was based on previous ERP findings (West and Ross-Munroe, 2002; West and Krompinger, 2005). Specifically, the amplitude of N300 over the occipital region (electrodes: O1/Oz/O2) was quantified and prospective positivity over the parietal region (electrodes: P3/Pz/P4) was measured.

Statistical comparisons were made at *p*-values of *p* < 0.05, with the Greenhouse–Geisser correction when violations of sphericity occurred.

## Results

### Behavioral Performance

Response times and accuracy in the two groups are presented in Table 2.

**Table 2:** **Accuracy (%) and response times (ms) for symptomatically remitted patients with schizophrenia and healthy controls**.

	Remitted schizophrenia (*n* = 20) (M ± SEM)	Healthy controls (*n* = 20) (M ± SEM)
Accuracy (%)
PM cue	80.10 ± 4.60	93.95 ± 1.47
Ongoing activity	96.15 ± 0.82	96.85 ± 0.95
Response times (ms)
PM cue	916.66 ± 47.18	780.89 ± 21.22
Ongoing activity	1235.18 ± 51.81	998.20 ± 37.58

*M, mean; SEM, standard error of mean*.

**Figure 1:**
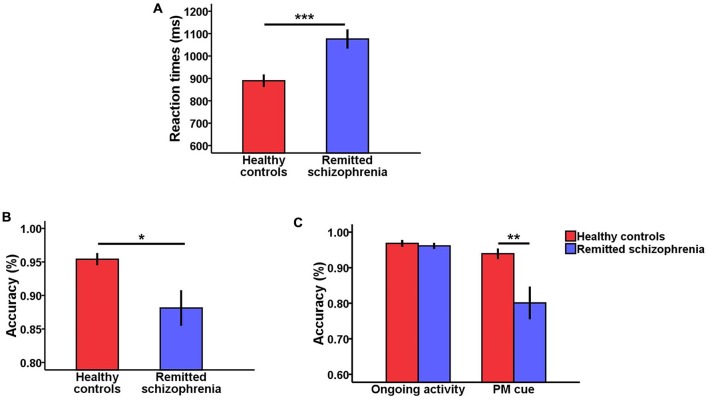
**Behavioral results. (A)** Bar plots of response times according to group (symptomatically remitted patients with schizophrenia vs. healthy controls). **(B)** Bar plots of accuracy according to group (symptomatically remitted patients with schizophrenia vs. healthy controls). **(C)** Bar plots of group × task type interaction effects on accuracy. Error bars indicate standard error of mean (SEM), (*p < 0.05, **p < 0.01, ***p < 0.001).

For response times, there was a significant main effect of group (*F*_(1,75)_ = 14.90, *p* < 0.001), revealing that symptomatically remitted patients with schizophrenia responded more slowly than healthy controls (Figure 1A). Moreover, there was also a significant main effect of task type (*F*_(1,75)_ = 42.91, *p* < 0.001), with response times being longer for ongoing activity trials than for PM cue trials. However, the interaction between group and task type was not significant (*F*_(1,75)_ = 1.53, *p* > 0.05).

With regard to accuracy, there was a significant main effect of group (*F*_(1,75)_ = 5.16, *p* < 0.05; Figure 1B). The symptomatically remitted patients with schizophrenia were less accurate than healthy controls, consistent with findings from a prior study using the similar task (Zhuo et al., 2013). Furthermore, accuracy was also significantly higher for ongoing activity trials than for PM cue trials (*F*_(1,75)_ = 14.71, *p* < 0.001). More importantly, we found a robust group × task type interaction (*F*_(1,75)_ = 7.10, *p* < 0.01). An analysis of simple effects showed that this interaction was driven by low accuracy on PM cue trials in symptomatically remitted patients with schizophrenia compared with healthy controls (*p* < 0.01; Figure 1C).

### ERP Results

#### N300

**Figure 2:**
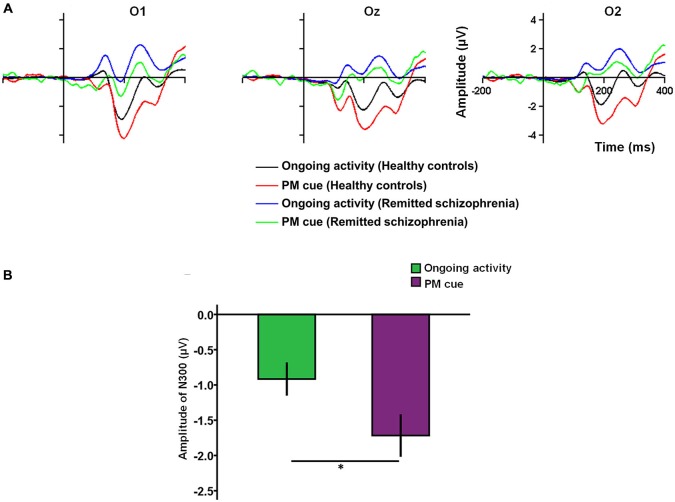
**The N300 related to prospective memory (PM) cue detection. (A)** The N300 over the occipital region (O1/Oz/O2) for PM cue trials and ongoing activity trials in symptomatically remitted patients with schizophrenia and healthy controls. **(B)** Bar plots illustrating the amplitudes of the N300 according to task type (PM vs. ongoing task). Error bars indicate SEM, (*p < 0.05).

Regarding the N300, there was a main effect of task type (*F*_(1,227)_ = 6.43, *p* < 0.05), with the N300 amplitude being more negativity for PM cue trials than for ongoing activity trials (Figures 2A,B). There was also a main effect of group (*F*_(1,227)_ = 89.24, *p* < 0.001), with the amplitude of the N300 being larger in healthy controls than in symptomatically remitted patients with schizophrenia. In addition, the main effect of electrode sites was also significant (*F*_(2,227)_ = 3.30, *p* < 0.05). The *post hoc* analysis revealed a significant difference between O2 and O1 (*p* < 0.05), with the amplitude of the N300 being larger in the electrode O2 than in the electrode O1. However, no significant interaction effects among these three factors were found.

#### Prospective Positivity

**Figure 3:**
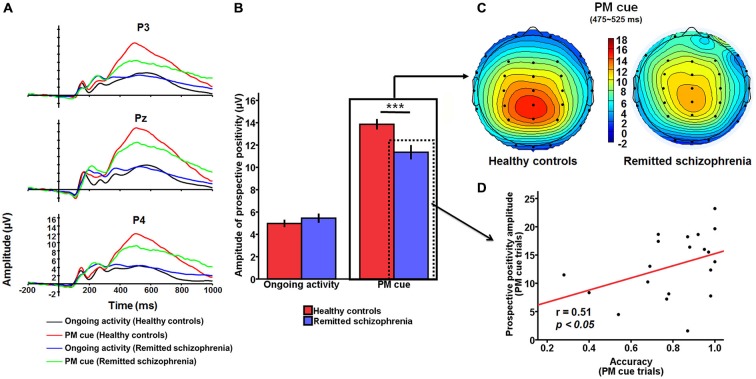
**The prospective positivity related to PM intention retrieval. (A)** The prospective positivity over the parietal region (P3/Pz/P4) for PM cue trials and ongoing activity trials in symptomatically remitted patients with schizophrenia and healthy controls. **(B)** Bar plots illustrating the group × task type interaction effect on the amplitude of the prospective positivity over the parietal region. As illustrated in the figure, this interaction effect is driven by the reduced amplitude of prospective positivity elicited by PM cues in symptomatically remitted patients with schizophrenia compared with healthy controls. Error bars indicate SEM, ***p < 0.001. **(C)** Topographical voltage distributions within 475–525 ms centered on the peak of prospective positivity elicited by PM cue for healthy controls (left) and symptomatically remitted patients with schizophrenia (right). Positive isopotential lines are in red and negative isopotential lines are in blue. As shown in the figure, there is reduced amplitude of prospective positivity over the parietal region in those patients compared with healthy controls. **(D)** Correlation between accuracy and amplitude of prospective activity on PM cue trials over the parietal region in remitted patients with schizophrenia. As illustrated in this scatter plot, amplitude of PM cue-elicited prospective amplitude over the parietal region is positively correlated with accuracy for PM cue trials in those patients, (*p* < 0.05).

For the prospective positivity, there was a significant main effect of group (*F*_(1,227)_ = 4.62, *p* < 0.05), with the amplitude of prospective positivity being larger in healthy controls than in symptomatically remitted patients with schizophrenia (Figure 3A). There was also a significant main effect of task type (*F*_(1,227)_ = 250.61, *p* < 0.001), with the amplitude being larger for the PM cue trials than for ongoing activity trials. In addition, there was a main effect of electrode sites (*F*_(2,227)_ = 5.44, *p* < 0.01). The *post hoc* analysis revealed a significant difference between Pz and two other electrode sites (P3 and P4; *p* < 0.05 and *p* < 0.005), indicating that prospective positivity was mainly distributed over the electrode (Pz) which was placed in the central location of the parietal region. More importantly, group × task type interaction was significant (*F*_(1,227)_ = 10.19, *p* < 0.01). An analysis of simple effects revealed that this interaction was due to smaller amplitude of the prospective positivity for PM cue trials in symptomatically remitted patients with schizophrenia than in healthy controls (*p* < 0.001; Figures 3B,C). Given the existence of interaction between group and task type for both accuracy and prospective positivity over the parietal region, it is interesting to investigate whether there is a link between accuracy on PM cue trials and PM cue-elicited prospective positivity in symptomatically remitted patients with schizophrenia. A partial correlation analysis, with IQ statistically controlled, revealed a significant positive correlation between them (*r* = 0.51, *p* < 0.05), suggesting that larger amplitude of prospective positivity was associated with better accuracy on the PM cue trials in symptomatically remitted patients with schizophrenia (Figure 3D).

## Discussion

The present study was to examine the ability to perform an intended action in symptomatically remitted patients with schizophrenia. The behavioral data revealed that symptomatically remitted patients with schizophrenia performed more poorly during the event-based PM task than healthy controls, which was driven by the decreased accuracy on PM cue trials in those remitted patients relative to healthy controls. This finding suggests that symptomatically remitted patients with schizophrenia are associated with PM deficits. Since there was a significant between-group difference in IQ and a number of studies revealed that intelligence was associated with PM performance in schizophrenia patients (Henry et al., 2007; Wang et al., 2009), this seemed to suggest that the observed PM deficits may be due to discrepancy in IQ between these two groups. However, the observed PM deficits in symptomatically remitted patients with schizophrenia were still maintained when IQ was statistically controlled, suggesting that PM deficits in symptomatically remitted patients with schizophrenia may be a primary deficit not secondary to other cognitive functions such as IQ. Despite such evidence, the causes of PM deficits in these remitted schizophrenia patients is not readily apparent merely on the basis of behavioral findings. It may arise from difference in the processes supporting the prospective component of PM between these two groups, differences in the processes supporting the retrospective component between these two groups or both. Our electrophysiological data may help shed important insights on it.

### ERP Related to Cue Detection

N300 over the occipital region has been demonstrated to be preferentially related to prospective component (cue detection) of PM (West et al., 2001; West and Ross-Munroe, 2002; West and Krompinger, 2005; West, 2011). It has been proposed that prospective component of PM reflects the biasing of attention between external events (e.g., identifying a cue amid distracting stimuli; Smith and Bayen, 2004). Our electrophysiological data showed that symptomatically remitted patients with schizophrenia were able to distinguish PM cues from the ongoing task as reflected by the significant main effect of task type across these two groups and the non-significant interaction between task type and group. As a consequence, this observation may indicate a degree of functional recovery of preparatory attentional processes that facilitates the processing of PM cues in these patients during clinical remission, thereby providing further evidence for recent studies showing that symptomatic remission in schizophrenia is associated with a degree of functional recovery of attentional processes (Fukumoto et al., 2014).

### ERP Related to Intention Retrieval

In contrast, our electrophysiological data related to retrospective component (intention retrieval) of PM showed that the amplitude of PM cue-elicited prospective positivity elicited was significantly decreased in symptomatically remitted patients with schizophrenia relative to healthy controls. This is corroborated by our subsequent observation about the positive correlation between such reduced prospective positivity and reduced accuracy on the PM task in symptomatically remitted patients with schizophrenia. Thus, these findings clearly demonstrate that PM deficits are characterized by the impairment in retrieving intentions from memory in remitted schizophrenia patients, which is distinct from acute episode of schizophrenia patients characterized by impairments in both cue detection and intention retrieval (Woods et al., 2007; Wang et al., 2008). This may have several important clinical implications. Our preliminary evidence about the selective deficit in PM intention retrieval in symptomatically remitted schizophrenia patients may have an impact on evaluation and improvement of the effectiveness of schizophrenia therapeutics during clinical remission because PM-based effective treatment interventions rely on precise characterization of PM deficits in symptomatically remitted patients with schizophrenia. As such, our finding may help direct clinicians to the selective deficit in retrospective component of PM and develop targeted psychological treatments and PM-based remediation strategies to improve functional outcomes in schizophrenia during clinical remission. Furthermore, there is recent evidence showing that PM can act as an important predictor of medication management ability and have an impact on medication adherence in acute phases of schizophrenia (Zogg et al., 2012; Lam et al., 2013), our observation in this study may indicate that the effective treatment interventions on the selective deficit in the retrospective component of PM may be one of the effective means of improving adherence in schizophrenia during clinical remission.

In addition, given that the selective deficit in retrospective component of PM in remitted schizophrenia patients is still observed in the present study and PM is thought to be a unique form of episodic memory (an important category of declarative memory; Kliegel et al., 2008), this seems to indicate that episodic memory deficit is one of the core impairments in schizophrenia patients and thus, may serve as one of stable markers of clinical remission in schizophrenia patients.

Taken together, these findings suggest a degree of functional recovery of processes associated with PM cue detection in symptomatically remitted schizophrenia patients, while the impairment in processes associated with PM intention retrieval still maintains.

### Potential Limitations

The present study is not without any limitations. First, only male patients with symptomatically remitted schizophrenia were involved in the current study. It remains unclear whether these findings could be generalized to female patients with remitted schizophrenia. This is important because it has been shown that gender differences exist in several aspects of schizophrenia (Ochoa et al., 2012). Second, one type of PM (event-based PM) was only involved in the present study. It is unknown whether these findings could be generalized to the other two types of PM (time-based and activity-based PM). This is important because recent research suggests that PM tasks cannot be viewed as being interchangeable and there are key differences among different types of PM tasks (Ordemann et al., 2014). Third, given that prior studies have shown that clinical remission can be predicted by the onset conditions of the schizophrenia patients (Marchesi et al., 2014), it should be interesting to link the deficits in cognitive functioning to the onset conditions of those remitted schizophrenia patients in the follow-up studies. Despite these limitations, we think that our current findings are still robust and may foster further research on neuropsychological characteristics of PM in symptomatically remitted patients with schizophrenia.

### Conclusion

In conclusion, the present study aimed at characterizing event-related potential correlates of PM in symptomatically remitted patients with schizophrenia. Our main finding is that on the neural level, the amplitude of the prospective positivity, a component of the ERPs related to PM intention retrieval, was significantly attenuated in symptomatically remitted schizophrenia patients relative to healthy controls. Moreover, the amplitude of this component was positively correlated with the accuracy on PM task in those patients. Therefore, this study demonstrates PM deficits in symptomatically remitted patients with schizophrenia characterized by a selective deficit in retrospective component of PM. This is distinct from PM deficits in acute phase of schizophrenia patients showing impairments in both prospective and retrospective components of PM. Our results may have important implications for further research charactering PM alterations in patients with symptomatically remitted schizophrenia.

## Author Contributions

GC, HC and YL designed the experiment; GC, LZ, WD, PX, SL, ZJ, LS, HL and HC performed the experiment; WD, PX, SL, ZJ, LS, HL analyzed behavioral data; GC, RZ and YL analyzed data; GC and YL wrote the manuscript.

## Funding

This work was supported by the Program of the “Twelfth Five-year Plan” for Science and Technology Research of China (grant number: AWS12J004 to HC) and Cultivation Program of Medical Science and Technique Program for Young Scientists (grant number: 14QNP006 to GC and YL).

## Conflict of Interest Statement

The authors declare that the research was conducted in the absence of any commercial or financial relationships that could be construed as a potential conflict of interest.

## References

[B1] Altgassen M. Kliegel M. Rendell P. Henry J. D. Zöllig J. (2008). Prospective memory in schizophrenia: the impact of varying retrospective-memory load. J. Clin. Exp. Neuropsychol. 30, 777–788. 10.1080/13803390701779552 18608664

[B2] Andreasen N. C. Carpenter W. T. Kane J. M. Lasser R. A. Marder S. R. Weinberger D. R. (2005). Remission in schizophrenia: proposed criteria and rationale for consensus. Am. J. Psychiatry 162, 441–449. 10.1176/appi.ajp.162.3.441 15741458

[B3] Association A. P. (2000). Diagnostic And Statistical Manual of Mental Disorders DSM-IV-TR Fourth Edition (Text Revision). Washington, DC: American Psychiatric Association.

[B4] Brandimonte M. A. Einstein G. O. Mcdaniel M. A. (2014). Prospective Memory: Theory and Applications. New York, NY: Psychology Press.

[B5] Chan R. Chen E. Cheung E. Cheung H. (2004). Executive dysfunctions in schizophrenia. Relationships to clinical manifestation. Eur. Arch. Psychiatry Clin. Neurosci. 254, 256–262. 10.1007/s00406-004-0492-3 15309397

[B6] Delorme A. Makeig S. (2004). EEGLAB: an open source toolbox for analysis of single-trial EEG dynamics including independent component analysis. J. Neurosci. Methods 134, 9–21. 10.1016/j.jneumeth.2003.10.009 15102499

[B7] Einstein G. O. McDaniel M. A. (1990). Normal aging and prospective memory. J. Exp. Psychol. Learn. Mem. Cogn. 16, 717–726. 10.1037/0278-7393.16.4.717 2142956

[B8] Elvevåg B. Goldberg T. E. (2000). Cognitive impairment in schizophrenia is the core of the disorder. Crit. Rev. Neurobiol. 14, 1–21. 10.1615/critrevneurobiol.v14.i1.10 11253953

[B9] Elvevåg B. Maylor E. Gilbert A. (2003). Habitual prospective memory in schizophrenia. BMC psychiatry 3:9. 10.1186/1471-244X-3-9 12890293 PMC184442

[B10] First M. B. Spitzer R. L. Gibbon M. Williams J. B. (2001). Structured Clinical Interview for DSM-IV-TR Axis I Disorders—Patient Edition (SCID-I/P. 2/2001 Revision). New York: Biometrics Research Department, New York State Psychiatric Institute.

[B11] Fukumoto M. Hashimoto R. Ohi K. Yasuda Y. Yamamori H. Umeda-Yano S.. (2014). Relation between remission status and attention in patients with schizophrenia. Psychiatry Clin. Neurosci. 68, 234–241. 10.1111/pcn.12119 24313598

[B12] Gong Y. (1992). Manual of Wechsler Adult Intelligence Scale-Chinese Version. Changsha: Chinese Map Press.

[B13] Graf P. Uttl B. (2001). Prospective memory: a new focus for research. Conscious. Cogn. 10, 437–450. 10.1006/ccog.2001.0504 11790035

[B14] Heinrichs R. W. Zakzanis K. K. (1998). Neurocognitive deficit in schizophrenia: a quantitative review of the evidence. Neuropsychology 12, 426–445. 10.1037/0894-4105.12.3.426 9673998

[B15] Henry J. D. Rendell P. G. Kliegel M. Altgassen M. (2007). Prospective memory in schizophrenia: primary or secondary impairment? Schizophr. Res. 95, 179–185. 10.1016/j.schres.2007.06.003 17630257

[B16] Jung T.-P. Makeig S. Humphries C. Lee T.-W. McKeown M. J. Iragui V.. (2000). Removing electroencephalographic artifacts by blind source separation. Psychophysiology 37, 163–178. 10.1111/1469-8986.3720163 10731767

[B17] Kay S. R. Flszbein A. Opfer L. A. (1987). The positive and negative syndrome scale (PANSS) for schizophrenia. Schizophr. Bull. 13, 261–276. 10.1093/schbul/13.2.261 3616518

[B18] Kliegel M. Mcdaniel M. A. Einstein G. O. (2008). Prospective Memory: Cognitive, Neuroscience, Developmental and Applied Perspectives. New York, London: Taylor and Francis.

[B19] Kumar D. Nizamie S. H. Jahan M. (2005). Event-based prospective memory in schizophrenia. J. Clin. Exp. Neuropsychol. 27, 867–872. 10.1080/13803390490919100 16183619

[B20] Kumar D. Nizamie S. H. Jahan M. (2008). Activity-based prospective memory in schizophrenia. Clin. Neuropsychol. 22, 497–506. 10.1080/13854040701396687 17853129

[B21] Lam J. W. Lui S. S. Wang Y. Chan R. C. Cheung E. F. (2013). Prospective memory predicts medication management ability and correlates with non-adherence to medications in individuals with clinically stable schizophrenia. Schizophr. Res. 147, 293–300. 10.1016/j.schres.2013.04.010 23631929

[B22] Lopez-Calderon J. Luck S. J. (2014). ERPLAB: an open-source toolbox for the analysis of event-related potentials. Front. Hum. Neurosci. 8:213. 10.3389/fnhum.2014.00213 24782741 PMC3995046

[B23] Luck S. J. Mathalon D. H. O’donnell B. F. Hämäläinen M. S. Spencer K. M. Javitt D. C.. (2011). A roadmap for the development and validation of event-related potential biomarkers in schizophrenia research. Bio. Psychiatry 70, 28–34. 10.1016/j.biopsych.2010.09.021 21111401 PMC3116072

[B24] Lui S. S. Wang Y. Liu A. C. Chui W. W. Gong Q.-Y. Shum D.. (2011). Prospective memory in patients with first-onset schizophrenia and their non-psychotic siblings. Neuropsychologia 49, 2217–2224. 10.1016/j.neuropsychologia.2011.04.002 21507327

[B25] Marchesi C. Affaticati A. Monici A. De Panfilis C. Ossola P. Tonna M. (2014). Predictors of symptomatic remission in patients with first-episode schizophrenia: a 16 years follow-up study. Compr. Psychiatry 55, 778–784. 10.1016/j.comppsych.2013.12.011 24461689

[B26] McDaniel M. A. Einstein G. (1992). “Aging and prospective memory: basic findings and practical applications,” in Advances in Learning and Behavioral Disabilities (Vol. 8), eds Scruggs T. E. Mastropieri M. A. (Greenwich, CT: JAI Press), 87–105.

[B27] Meacham J. A. Singer J. (1977). Incentive effects in prospective remembering. J. Psychol. 97, 191–197. 10.1080/00223980.1977.9923962

[B28] Mesholam-Gately R. I. Giuliano A. J. Goff K. P. Faraone S. V. Seidman L. J. (2009). Neurocognition in first-episode schizophrenia: a meta-analytic review. Neuropsychology 23, 315–316. 10.1037/a0014708 19413446

[B29] Mo S. Su Y. Chan R. C. Liu J. (2008). Comprehension of metaphor and irony in schizophrenia during remission: the role of theory of mind and IQ. Psychiatry Res. 157, 21–29. 10.1016/j.psychres.2006.04.002 17854910

[B30] Ochoa S. Usall J. Cobo J. Labad X. Kulkarni J. (2012). Gender differences in schizophrenia and first-episode psychosis: a comprehensive literature review. Schizophr. Res. Treatment 2012, 1–9. 10.1155/2012/916198 22966451 PMC3420456

[B31] Ordemann G. J. Opper J. Davalos D. (2014). Prospective memory in schizophrenia: a review. Schizophr. Res. 155, 77–89. 10.1016/j.schres.2014.03.008 24698096

[B32] Phillips S. M. Sherwin B. B. (1992). Variations in memory function and sex steroid hormones across the menstrual cycle. Psychoneuroendocrinology 17, 497–506. 10.1016/0306-4530(92)90008-u 1484916

[B33] Reichenberg A. Harvey P. D. (2007). Neuropsychological impairments in schizophrenia: integration of performance-based and brain imaging findings. Psychol. Bull. 133, 833–858. 10.1037/0033-2909.133.5.833 17723032

[B34] Rendell P. G. Henry J. D. (2009). A review of virtual week for prospective memory assessment: clinical implications. Brain Impair. 10, 14–22. 10.1375/brim.10.1.14

[B35] Shum D. Ungvari G. S. Tang W.-K. Leung J. P. (2004). Performance of schizophrenia patients on time-, event- and activity-based prospective memory tasks. Schizophr. Bull. 30, 693–702. 10.1093/oxfordjournals.schbul.a007123 15954184

[B36] Smith R. E. Bayen U. J. (2004). A multinomial model of event-based prospective memory. J. Exp. Psychol. Learn. Mem. Cogn. 30, 756–777. 10.1037/0278-7393.30.4.756 15238021

[B37] Ungvari G. S. Xiang Y.-T. Tang W.-K. Shum D. (2008). Prospective memory and its correlates and predictors in schizophrenia: an extension of previous findings. Arch. Clin. Neuropsychol. 23, 613–622. 10.1016/j.acn.2008.06.005 18635339

[B38] Wang Y. Chan R. C. Hong X. Ma Z. Yang T. Guo L.. (2008). Prospective memory in schizophrenia: further clarification of nature of impairment. Schizophr. Res. 105, 114–124. 10.1016/j.schres.2008.07.002 18707848

[B39] Wang Y. Cui J. Chan R. C. Deng Y. Shi H. Hong X.. (2009). Meta-analysis of prospective memory in schizophrenia: nature, extent and correlates. Schizophr. Res. 114, 64–70. 10.1016/j.schres.2009.07.009 19713081

[B40] West R. (2011). The temporal dynamics of prospective memory: a review of the ERP and prospective memory literature. Neuropsychologia 49, 2233–2245. 10.1016/j.neuropsychologia.2010.12.028 21187107

[B43] West R. Herndon R. W. Crewdson S. J. (2001). Neural activity associated with the realization of a delayed intention. Brain Res. Cogn. Brain Res. 12, 1–9. 10.1016/s0926-6410(01)00014-3 11489603

[B41] West R. Krompinger J. (2005). Neural correlates of prospective and retrospective memory. Neuropsychologia 43, 418–433. 10.1016/j.neuropsychologia.2004.06.012 15707617

[B42] West R. Ross-Munroe K. (2002). Neural correlates of the formation and realization of delayed intentions. Cogn. Affect. Behav. Neurosci. 2, 162–173. 10.3758/cabn.2.2.162 12455683

[B44] Wizeman T. M. (2012). Sex-Specific Reporting of Scientific Research: A Workshop Summary. Washington, DC: National Academies Press. 22379657

[B45] Woods S. P. Twamley E. W. Dawson M. S. Narvaez J. M. Jeste D. V. (2007). Deficits in cue detection and intention retrieval underlie prospective memory impairment in schizophrenia. Schizophr. Res. 90, 344–350. 10.1016/j.schres.2006.11.005 17175138 PMC1851918

[B46] Xiang Y.-T. Shum D. Chiu H. F. Tang W.-K. Ungvari G. S. (2010). Independent association of prospective memory with retrospective memory and intelligence in schizophrenia: a controlled study. Arch. Clin. Neuropsychol. 25, 680–684. 10.1093/arclin/acq062 20716544

[B47] Yalcin-Siedentopf N. Hoertnagl C. M. Biedermann F. Baumgartner S. Deisenhammer E. A. Hausmann A.. (2014). Facial affect recognition in symptomatically remitted patients with schizophrenia and bipolar disorder. Schizophr. Res. 152, 440–445. 10.1016/j.schres.2013.11.024 24361305

[B48] Zhou F.-C. Xiang Y.-T. Wang C.-Y. Dickerson F. Au R. W. Zhou J.-J.. (2012). Characteristics and clinical correlates of prospective memory performance in first-episode schizophrenia. Schizophr. Res. 135, 34–39. 10.1016/j.schres.2011.12.001 22222379

[B49] Zhuo K. Lu Y. Yang Z. Fan X. Song Z. Liao L.. (2013). Prospective memory performance in patients with drug-naïve, first-episode psychosis. Schizophr Res. 143, 285–290. 10.1016/j.schres.2012.12.002 23267733

[B50] Zogg J. B. Woods S. P. Sauceda J. A. Wiebe J. S. Simoni J. M. (2012). The role of prospective memory in medication adherence: a review of an emerging literature. J. Behav. Med. 35, 47–62. 10.1007/s10865-011-9341-9 21487722 PMC3574793

